# Supplementary material for: Are carbon emissions trading and green financial instruments synergistic? -Comprehensive quantitative research based on content analysis
Source: PLoS One. 2024 Mar 7;19(3):e0298601. doi: 10.1371/journal.pone.0298601 (PMC10919601; doi:10.1371/journal.pone.0298601)
Supplement: S1 Data — (ZIP) [file pone.0298601.s001.zip › Data/地级市Z_F工作报告.zip!/╜¡╬≈/╛┼╜¡╩╨/└┤╫╘╣1⁄2╓┌║┼í╛┬φ┐╦╩2╛▌═°í┐_2007.docx]

各位代表：

　　我代表市人民政府，向大会作工作报告，请予审议，并请各位政协委员和列席会议的同志提出意见。

一、过去三年工作回顾

三年来，在省委、省政府和市委的正确领导下，我们高举邓小平理论和“三个代表”重要思想伟大旗帜，认真贯彻落实科学发展观，紧紧依靠全市人民，开拓进取，扎实工作，经济和社会发展取得了新成就。

经济持续较快增长。如期完成“十五”计划各项任务，２００６年，预计全市生产总值５０６．８５亿元，年均增长１５．２％。财政总收入４６亿元，年均增长１７．２％；地方财政收入２４．６３亿元，年均增长１７．３２％。规模以上工业增加值１２６亿元，规模以上工业企业达到５００户，产值过亿元企业６０户，利税超亿元企业新增５户。实现了县县都有工业园，入园企业达１２０７个，园区工业占全市工业增加值的２８％。农业增加值７８亿元，年均增长６．２％；产业规模不断壮大，产业特色更加明显，建立无公害农产品生产基地８０个，培育国家和省级龙头企业２１家，农产品优质品率达９０％以上。累计完成城镇固定资产投资５６１．２２亿元，年均增长２４％。社会消费品零售总额１３９亿元，年均增长１７．５％；全社会货运总量３２００万吨，增长２６％；集装箱吞吐量达８万标箱，增长４３％；十大商场销售额和十大市场成交额分别增长３０％和２８％。

改革开放深入推进。市属国有、集体企业改制完成２６户。部门预算、国库集中支付、乡财县管等财政综合改革全面推行。农村税费改革实现了农民合同内零负担。林权制度改革稳步推进。机构改革和４５家企业学校剥离工作全面完成。出口加工区申报成功，通过验收。共青开发区被评为中国羽绒服装城。连续三年成功承办赣台经贸合作研讨会。累计引进外资项目４２２个，引进内资项目３９１４个，新上亿元以上项目８３个。外贸出口年均增长４４．４％。民营经济发展加快，上缴税收占全市财政总收入３８．０４％。庐山管理体制进一步理顺，云居山—柘林湖申报批准为国家重点风景名胜区并实现统一管理，旅游经济出现转折性变化，国内游客突破１０００万人次，入境游客突破１０万人次。海关、检验检疫、外汇管理等驻市机构和外事侨务、对台事务、驻外机构、商会等在对外开放中发挥了积极作用。

城乡面貌明显改观。完成了城市总体规划、市域城镇体系规划以及２０多个专项规划，中心城区控制性规划覆盖率达９５％。加强国有资产监管，城市公共资本市场化运作取得良好效益。市区基础设施建设累计投资达２５亿元，建成区面积扩大到７０平方公里。新增城市道路４０公里、管网２００公里、绿地２００万平方米，建成应急救援指挥中心、老年和少儿活动中心、三水厂二期扩建、白水明珠、滨江生态园等一批功能公益性设施。城市管理有效机制基本形成，市容市貌焕然一新。大气污染治理富有成效，环境质量明显改善。２００４年获得中国魅力城市称号。环庐山、修武南线等公路和九武铁路复线建成通车，铜九铁路、武吉高速公路建设进展顺利。５０万伏输变电工程开工建设。原油输油管线九江段已投入使用。新农村建设开局良好，农民生产生活基础设施建设投入累计达２亿元。乡村公路建设完成３９５１公里，农村电网改造基本完成。县域经济快速发展，财政收入实现县县过亿元，其中５个超２亿元，４个突破３亿元。

社会事业全面进步。公共事业投入不断加大，经济社会发展更加协调。科技兴市稳步推进，获得国家科技进步奖４２项，申请专利３１３件。“两基”教育覆盖率达１００％，完成校舍危房改造３３．２７万平方米，高校在校生达９．５６万人，民办学校在校生达９．９１万人。市图书馆建成开放，农村科技、卫生、文化三项活动全面铺开。新型农村合作医疗试点工作顺利推进，疾病预防控制体系基本建立，结核病、血吸虫病综合治理有所加强。群众体育运动蓬勃开展，竞技体育水平进入全省前列。广播电视覆盖范围扩大。人口性别比得到优化，人口素质不断提高。新闻出版、妇女儿童、档案、残联、民族宗教、老龄等工作取得新的进展。

人民生活进一步改善。预计城镇居民可支配收入９５９０元，年均增长１０．８％；农民人均纯收入３５６９元，年均增长１４．６％。“十大民心工程”、“百件惠民实事”解决了一些关系群众切身利益的问题。养老、失业、医疗保险覆盖面不断扩大，累计新增就业１７．７５万人，６．６６万人实现再就业。残疾人康复中心、福利院、敬老院等社会救助体系建设基本满足了特殊群体的需要，农村“五保”集中供养率达７１％。市区居民人均住房建筑面积达２６．３１平方米，首期廉租房交付使用。农村学生自带课桌椅问题基本解决。农垦、老建扶贫、库区和深山移民搬迁工作有新进步，防汛抗旱、气象、水文、地震、地质、人防等单位在防灾减灾中作出积极贡献，粮食、物资、供销、医药、盐业、烟草等行业在服务人民生产生活方面有新提高。

依法行政步伐进一步加快。深入开展保持共产党员先进性教育活动，贯彻落实《行政许可法》和《公务员法》。累计办理行政复议１８７件，取消审批事项８７项。政务公开和行政部门公开承诺制全面推行，建立政府信息公开制度，执法监察和行风评议深入开展。严肃查处影响投资环境和损害群众利益的案件。“四五”普法完成。始终保持严打高压态势，社会治安综合治理得到加强。国防教育和后备力量建设稳步发展，连续被评为全省“双拥模范城”。驻浔人民解放军、武警官兵在急难险重的关键时刻发挥了重要作用。依法接受人大监督和政协民主监督，累计办理人大代表建议和政协委员提案１６５０件。加大信访突出问题解决力度。统计、审计、工商、国税、地税、物价、安监、质监、药监等部门在规范市场经济秩序中取得新成绩。

总体看，这三年改革深化、发展提速、城乡进步、社会稳定。我们适应宏观调控新形势，破解了一批制约经济社会发展的难题，实现了经济发展持续快速增长；我们完成了一批重点设施和重大项目建设，经济发展后劲明显增强；我们倾心尽力为群众办实事，人民群众的生产生活条件逐步改善。特别在强台风、地震等严重自然灾害面前，我们临危不乱，沉着应对，万众一心，顽强拼搏，迅速恢复生产，重建家园，展示了九江人民的崭新形象。这些成绩的取得，是省委、省政府和市委正确领导的结果，是市人大、政协监督支持的结果，是全市人民同心同德、团结奋斗的结果。在此，我代表市人民政府，向全市工人、农民、知识分子和广大市民，向各位人大代表、政协委员，向各民主党派、工商联、无党派人士、各人民团体和驻市单位，向驻浔人民解放军、武警官兵和公安干警，向所有关心、支持九江发展的各界朋友，表示衷心的感谢和诚挚的敬意！

我们也清醒地看到，当前我市经济社会发展中还存在着一些困难和问题。主要是经济总量还不大，发展不够仍是主要矛盾，与江西北港重镇地位还不相称；财政实力还不强，财源结构比较单一；增长方式比较粗放，主导产业竞争力不强；就业、社保压力仍然较大，部分群众生活还很困难；体制、机制活力不够，内生经济活力不足；少数机关干部作风不实、效率不高、服务不优的问题还比较严重，发展环境有待进一步改善。我们一定采取有效措施，切实加以解决。

二、今后五年的工作目标和主要任务

今后五年，是实现九江经济社会又好又快发展的关键时期。放眼全球，国际产业加速向我国转移，科技革命方兴未艾；纵观国内，中部崛起战略深入实施，区域合作不断拓展；立足九江，全省发展呈现良好态势，我们自身积蓄的发展能量持续释放，历届班子奠定了良好工作基础。这些机遇的聚合，为我们实现又好又快发展创造了十分有利的条件。在新的起点上，坚决贯彻落实中共九江市第九次代表大会精神，抓住机遇，发挥优势，凝心聚力，富民兴市，是我们义不容辞的责任，也是全市人民的共同愿望。

今后五年，我们要以邓小平理论和“三个代表”重要思想为指导，坚持以科学发展观统领全局，把构建社会主义和谐社会贯穿于发展的全过程，围绕建设美丽富裕和谐强盛的社会主义新九江目标，按照打造新型工业重镇、区域物流中心、文化旅游胜地、开放港口城市的要求，坚定实施大开放主战略，大力推进新型工业化和经济市场化，扎实开展社会主义新农村建设，不断加快农业农村现代化和新型城镇化和经济国际化，在更高层次上推进“３＋１”发展战略，推动经济社会发展再上新台阶！

经过五年的奋斗，努力实现以下经济社会发展目标：

——全市生产总值突破１０００亿元，年均增长１４％，人均ＧＤＰ突破２２００美元；财政总收入达到１００亿元，年均增长１６％，地方一般预算收入达到６０亿元，年均增长１８％；工业增加值占全市生产总值的比重达到５０％；累计利用外资３０亿美元，年均增长１５％；实际利用市外资金年均增长１５％；外贸出口年均增长２５％；全社会固定资产投资累计完成２８００亿元，年均增长３０％；社会消费品零售总额年均增长１４％。

——人口自然增长率控制在８‰以内；科技进步对经济增长的贡献率达到６０％；普及九年制义务教育人口覆盖率达到１００％，高中阶段教育普及率达到９０％，高等教育毛入学率达到２５％；公共卫生和基本医疗服务进一步加强，实现全市千人拥有医院病床数２．４５张，千人拥有医生数１．５１人。

——城镇居民人均可支配收入达到１４０００元；农民人均纯收入达到５２６５元；城镇登记失业率控制在４％以内，累计新增农村劳动力转移４０万人，新增城镇就业４０万人；城镇养老、医疗、失业保险覆盖率及新型农村合作医疗覆盖率分别达到８０％、８２％、７６％和８０％以上，城镇人均住房建筑面积达到３０平方米。

——单位ＧＤＰ能耗降低２０％以上；主要污染物排放总量减少２０％以上；工业固体废弃物综合处理率达到９６％；城市污水集中处理率达到７０％；生活垃圾无害化处理率达到７０％；森林覆盖率达到５２％以上；集中饮用水源水质达标率１００％。

——城市建成区面积１００平方公里，城市人口１００万，城镇化率达到５０％，中心城区空气环境质量优于Ⅱ类标准，争创全国文明卫生城和“中国宜居城市”。

今后五年，重点在以下六个方面奋力推进，取得实实在在的成效。

（一）提高对外开放水平，增强创新创业活力

坚持以开放借外力，以创业添活力，以改革增动力，不断提升经济的国际化市场化水平。

加大利用外资力度，努力提高经济外向度。积极对接全球市场，参与国际分工；承接产业转移，推进区域合作；打造毗邻经济圈，构筑省际开放桥头堡。进一步解放思想，坚持以观念接轨为先导、交通接轨为基础、产业接轨为核心、市场接轨为依托、企业接轨为主体，在承接中提升，在竞争中合作，在互补中共赢。坚持以项目换投资，以存量引增量，重点加大对跨国公司特别是世界５００强和国内大企业、大集团的招商力度，扩大对港澳台、东南亚、欧美日韩等资本、技术的引进。不断拓展对外开放领域，引导外资投向现代制造业、现代服务业和现代农业。坚持以环境招商、以产业招商、以比较优势招商，依托工业园区和出口加工区，围绕产业集群、产业配套，着力引进投资规模大、技术含量高、带动能力强的重大项目。积极发展对外贸易，转变外贸增长方式，扩大出口产品的技术和价值含量，培植优势产品出口生产基地。讲求对外开放的综合效益，在矿产开发上，鼓励精深加工，防止资源的粗放开发，切实保护环境；在土地利用上，鼓励集约型、节约型项目，防止土地闲置浪费。

大力推进全民创业，加快民营经济发展。着力激活创业主体，鼓励大中专毕业生、转业军人及下岗工人自主创业，退伍士兵及农村基层干部群众致富创业，外出务工人员返乡创业，科技人员兴办实体创业，留学生归国创业。着力拓宽创业领域，激发内生经济的活力。凡是对外资开放的投资领域，一律向民营资本开放。既鼓励以劳动和资本等有形要素创业，也鼓励以知识、技术、技能、管理等无形要素创业；既鼓励创办和发展各种实业，也鼓励支持非实业形态的创业。着力完善创业政策，设立创业基金，搞好创业培训，优化创业服务，扩大小额贷款政策覆盖面，改善民营企业发展的融资环境。着力培育创业文化，营造政府鼓励创业、社会支持创业、民众自主创业的良好发展氛围。

进一步深化改革，推进体制机制创新。深化国有资产监管体制改革和产权多元化改革，加快建立现代企业制度，健全法人治理机制，鼓励和支持国有企业通过改革重组，整合资源，靠大联强。树立资本经营理念，积极运作符合条件的企业上市。加快推进市场体系建设，促进资源配置市场化。进一步转变政府职能，规范政府行为，建立健全政府决策机制、执行机制、推进机制、监督机制以及应急机制。树立大财政理念，完善公共财政管理体制，进一步理顺市、县两级财政运行机制，优化财政收支结构，做到生财有道、聚财有方、理财有法、用财有度。深化投融资体制改革。巩固农村税费改革和林权制度改革成果，加快推进乡镇机构、农村义务教育和县乡财政管理体制等改革。进一步推进公用事业体制改革，分类推进事业单位改革，创新社会事业和保障运行机制。

优化发展环境，营造良好的发展氛围。无论是发展外向型经济，还是激励全民创业，环境都是关键。以政务环境建设为重点，把“亲商、安商、富商”措施落实到位。公平对待，一视同仁，兑现政策，履行承诺，营造诚信透明的政策环境；减少环节，集中审批，限时办结，一站服务，营造高效便捷的办事环境；多放少限，多帮少卡，多奖少罚，多予少取，营造开明有序的商务环境；深化审批制度改革，规范执法行为，全面推进依法行政，营造公平公正的法制环境。

（二）着力转变经济增长方式，加快新型工业化进程

坚持以新型工业化为核心，做大工业经济总量，形成工业为主导的经济结构，推进产业结构优化升级，不断提高经济增长的质量和水平。

优化工业布局，大力培育主导产业和产业集群。充分发挥长江岸线和昌九沿线的优势，主动承接武汉、南昌和长珠闽地区的辐射，加快沿江产业开发和昌九工业走廊建设，形成“Ｔ”型工业布局，努力建设长江中下游结合部区域现代制造业基地，形成临港工业带。重点建设码头、沙河、金砂湾、星火四大工业城，加快全市十二个工业园区建设，着力建设九江经济技术开发区、共青开发区和出口加工区。围绕石油化工、机械电子、冶金建材、纺织服装、电力能源、食品加工等六大支柱产业，大力调整工业产业结构，注重培育新的支柱产业。引导已有一定比较优势的石化、有机硅、化纤、玻纤、纺织、汽车、船舶、建材等产业延伸产业链，同时大力发展采矿及矿产品精深加工业，支持优势企业和支柱产业做大做强。

提升工业园区建设水平，增强增长极的带动作用。强化工业园区的主平台地位，完善园区基础设施，增强园区产业配套、集聚发展的能力。完善工业园区的服务体系，创新工作机制，提升管理水平。提高园区单位面积投资强度和投入产出效益，提高集约水平。加大对九江经济技术开发区、共青开发区、出口加工区的支持力度，进一步下放权力，激发活力，增强动力，努力建成全市工业发展的新平台。突出园区产业特色，促进各工业园依据自身的产业基础、区位条件、资源优势和环境特点适当的进行产业分工，注重与城区建设和县域经济发展相结合，提高集聚力、辐射力和带动力，形成各具特色的发展格局。

做优做强骨干企业，提升产业竞争实力。把建设大项目作为发展大企业的重要途径，扶优扶强提升现有大企业。招商引资多选大项目，支持县区增多大项目。积极靠大联强，通过改制盘活存量。优化产业组织结构，以市场为导向、资本为纽带，培育成长性强的骨干企业。围绕产业集群式发展，推动产业协作配套，促进大中小企业协调发展。加快传统产业改造，加快以信息技术为代表的高新技术和先进适用技术的扩散与渗透，提升传统产业的技术装备水平、生产工艺水平和产品质量档次。加快发展高科技循环经济产业，加快高技术研发及孵化，力争在电子信息、生物医药、新材料、计算机软件等重点领域取得新突破。组织专门力量研究品牌战略，保护知识产权，重视科技创新，提升品牌质量，扩大品牌效应，五年内品牌企业和品牌产品要有新突破。

（三）加快社会主义新农村建设，促进城乡统筹发展

坚持把社会主义新农村建设作为统揽农村经济社会发展的总抓手，按照中央“二十字”方针和省委“五新一好”的要求，建立“政府主导、农民主体、干部服务、社会参与”的工作机制，整合领导、部门、政策、措施及社会力量，调动农民群众的积极性、主动性，以发展农村经济和农民增收为核心，以村庄规划和“三清三改”为切入点，大力推进社会主义新农村建设，加快形成以工补农、城乡互动、协调发展的新型城乡关系。

从广大农民最盼望解决的问题入手，大力建设新村镇。坚持村镇规划先行，加快村镇规划编制，优化小城镇及村庄布局。以“走平坦路，喝干净水，上卫生厕，住整洁房”为重点，加大对农村基础设施建设支持力度，着力改善农村生产生活条件和居住环境，激发农民热爱家乡、建设家乡、发展经济的内在力量，力争五年内完成７０％的新村庄建设。新建和改造农村公路６４８０公里，基本实现广播电视村村通，电话基本“进村入户”；解决６５万人饮水安全，农村自来水普及率达到８０％；积极发展农村清洁能源，新增户型沼气池１２万个，农村无害化卫生厕所普及率达到５０％。

抓住农业发展、农民增收的关键环节，加快发展新产业。立足产业基础和资源优势，加强农业资源尤其是山水资源的综合开发，做大做强主导产业，培育特色产业基地。发展农产品精深加工，培育一批具有比较优势、带动力强的龙头企业，加强农民经济合作组织培育，不断提高农业产业化水平。实施农产品绿色品牌战略，完善农业支持保护体系，提高农业机械化水平，加强农业科技推广和农业标准化建设。实施严格的耕地保护制度，建设动植物防疫体系，加强小型农田水利建设，全面完成病险水库除险加固。

提升农村发展最活跃的因素，切实培育新农民。以培养新型务工农民和产业农民为目标，加强务工职业技能和农业实用技术培训，切实提高农民科技致富能力、市场竞争能力和自主发展能力。加强农民的思想道德、民主法制、文明卫生教育，培养有文化、讲道德、守法纪、懂技术、会经营的新型农民。加强农村基层政权建设，推进村务公开、政务公开和民主管理，完善乡村治理机制和村民自治，提高农民群众自我教育、自我服务、自我管理的水平。

营造和谐稳定的农村社会环境，全面塑造新风貌。加快农村社会事业发展，大力改善农村中小学办学条件，提高农村义务教育质量。改善农村居民的医疗卫生条件，加大对困难农民的救助。深入推进文明村镇创建活动，加强农村精神文明建设，引导农民树新风、破陋习，陶冶情操，告别贫困，迈向文明，营造和谐温馨稳定的人文和社会环境。

（四）加快开放港口城市建设步伐，努力提高城市综合竞争力

加快港口建设，发展现代物流，彰显文化魅力，打造旅游胜地，实现港口兴市、商贸活市、旅游旺市。

加强城市建设和管理，完善城市功能。推进中心城市建设，以产业发展、增加就业、人口集聚和相关城区形态的形成，构建区域中心城市的基本框架。以沿江、沿路、环山、环湖区域为重点，统筹城市空间布局，重视瑞昌、沙河、星子、湖口卫星城建设，加快县城和实力强的中心镇建设步伐，把有条件的县城建设成功能比较完善、人口和产业集聚能力较强的中小城市，构建以中心城市为龙头、中小城市共同发展的城镇体系。依据山、江、湖生态多样性的地域特色和建设生态九江的要求完善规划。在城市风格上彰显山水灵性，在人居环境中体现人与自然和谐，在城市人文上传承历史文化，努力塑造最适宜人居住的山水生态港口旅游城市。顺应港口开发和产业结构调整趋势，大力发展商贸、会展、金融保险、中介、法律、广告、现代物流等生产服务业，有序繁荣饮食休闲、娱乐健身、物业、社区服务等生活服务业。加快城市基础设施和公用事业的发展。重视传承和弘扬历史文化，有效保护修缮和恢复城市历史街区和古建筑，争创中国历史文化名城。进一步完善教育、文化、卫生、体育等功能设施，大力发展广播影视、演出艺术、体育等文化产业。创新城市管理机制，依法加强和规范城市管理。

发展现代物流，增强辐射能力。重视长江岸线港口规划，加快九江、金砂湾、官湖三大港区扩能扩容建设。加快构筑物流基础设施和信息平台，加快建设物流园区、物流中心、物流市场。加快大型专业市场建设，整合现有物流资源，引进外来物流企业，形成若干具有现代功能和运营模式的物流企业集团。大力促进铁、公、水、空等各种运输方式的有效衔接，努力构建交通运输、仓储配送、流通加工、信息网络等功能完善的现代物流服务体系，把九江建成辐射赣、鄂、皖、湘的区域性转港贸易物流中心。

发挥资源优势，壮大旅游产业。树立大旅游观念，全面整合九江“著名山水、避暑胜地、候鸟王国、宗教圣地”的独有优势，重点打好“庐山牌”，形成山带市、市助山的大旅游格局，形成九江旅游新优势。高起点策划，大手笔构建，推出一批内涵丰富、特色鲜明、品位高雅的旅游线路，建设一批精品旅游点。坚持吃住行游购娱并举，加强旅游产品开发，发展相关产业，提高旅游综合效益。坚持多元投入，培养旅游人才，完善旅游设施，规范旅游市场，提升服务质量。加强与周边旅游景区和旅游发达地区的合作，吸引国内外知名旅行社入驻经营，以沿海、沿江、沿京九线为重点，以“巩固港、澳、台，主攻日、韩、新，拓展欧、美、澳”为方向，不断拓宽境外市场空间。

发展县域经济，增强基层活力。支持各县（市、区）发挥优势，各展所长，壮大县域经济。以招商引资和发展龙头企业为抓手，以发展农产品加工业、大工业配套产业和地方特色工业为重点，主攻民营经济，壮大园区经济，做强特色经济，发展劳务经济，大力推进工业化、城镇化和农业产业化，实现富县富乡富民。进一步简政放权，赋予县乡、开发区和工业园更大的自主权和决策权，建立县级财政增长的激励机制，增强县域经济加快发展能力，争取实现１～２个县进入全省１０强行列。

（五）夯实发展基础，切实增强可持续发展的能力

进一步完善基础设施，繁荣教育科技事业，加快科技创新，加强环境保护，全面增强可持续发展能力。

加大基础设施建设力度，增强基础设施保障能力。依托长江黄金水道、京九铁路及高速公路建设立体交通网络，努力成为辐射带动功能较强的２小时城市经济圈和融入长江经济带、对接长珠闽的桥头堡。建设长江二桥、武吉高速、杭瑞高速（九江段），做好瑞昌、彭泽长江大桥前期工作，推进沿江快速通道建设，形成覆盖面较广、运载能力较强的高速公路网络。县至重点乡镇通二级以上公路，乡及行政村基本通油路或水泥路，全面提高路网通达度与等级。继续完善铁路网络，建成昌九城际轨道交通、铜九铁路，建设九景衢铁路。发展长江航运，构筑水运网络，加快形成长江沿线１５０万国际集装箱吞吐能力的战略布局，加快天然气储运码头的建设，构建全市快速便捷的综合交通体系。按照适度超前的原则加大能源建设力度，争取尽早启动九江电厂四期，尽快建成星子大岭、都昌老爷庙风电项目，积极推进彭泽核电建设项目，加紧原油管线、天然气管线、成品油管线的建设，建设九江——南昌——樟树成品油管线工程。加强各类水利设施建设，提高调洪防洪能力，全面完成防汛指挥系统和防汛通讯系统建设。提高城乡供水能力，抓好城市排水系统配套工程建设。加快信息化进程，以政府信息化带动社会信息化，提升国民经济、社会管理的水平和效率。拓宽基础设施投入渠道，抓住中央加大对中部地区交通、能源等基础设施投入的机遇，积极向上争取项目和资金，鼓励支持外资和民间资本参与基础设施建设。

加大投入力度，加快教育科技事业发展。优先发展教育事业，以农村为重点，巩固提高义务教育水平，切实提高高等教育质量，大力发展职业教育，抓紧培养专业化技能型人才和农村实用人才。提高市民的科技文化素质，努力建设学习型社会。加快科技创新，大力推进产学研结合，推动高新技术和先进技术在生产中的应用，通过政策引导鼓励企业加大技术创新投入，推进技术成果转化，形成一批具有较高科技含量、市场竞争力强的技术和产品。重视发挥人才在创新发展中的关键作用，加快建立与市场经济体制配套、与经济社会发展目标相适应、政府调控与市场调节相结合的人才资源开发体系，不断增加人才数量、提高人才质量、调整人才分布、改善人才结构，使人才引得进、留得住、用得好。

加强资源节约和环境保护，促进人口、资源、环境与经济社会发展相协调。进一步完善庐山生态功能区的保护，切实保护鄱阳湖湿地。加大森林资源保护和平原绿化力度，有计划实施封山育林，扩大公益林面积；有序开发经济林，实行限量采伐。严格控制林木加工项目，对其中技术先进、综合效益好的项目，先建造林基地，后布点加工。矿山开采实行植被强制恢复制度，控制水土流失。加强生态环境执法管理，按照“谁开发谁保护、谁受益谁补偿”的原则，加快建立生态补偿机制。重点加强鄱阳湖、修河、长河、博阳河流域和交通干线两侧、大中型水库、重要城镇周围、矿山等生态环境脆弱地区的环境治理和建设。坚持综合治理，加大大气污染、水污染和固体废弃物污染的防治力度，加快对污染严重的化工、印染、选矿、电镀、建材等行业的技术改造和治理，强制淘汰消耗高、污染重、技术落后的生产工艺和产品生产，提高城市污水处理能力。尽快完善促进资源节约的政策体系，建立健全使用资源监管体系。引导在现代农业、石油化工、能源产业方面发展循环经济，实现经济发展低消耗、低排放、高效益。认真执行计划生育基本国策，推进优生优育，提高人口素质，促进人口、资源、环境与经济社会协调发展。

（六）坚持以人为本，促进和谐社会建设

把解决民生问题当作政府的第一事务，妥善协调处理社会利益关系，促进社会和谐稳定，增进人民群众的幸福感。

实施积极的就业政策，进一步做好就业再就业工作。强化政府促进就业职能，完善政府扩大就业目标考核机制，统筹做好城镇新增劳动力就业、农村富余劳动力转移就业、下岗失业人员再就业工作，加强对大中专毕业生、退役军人就业指导工作。多渠道、多方式增加就业岗位，大力发展劳动密集型产业、服务业、非公有制经济和中小企业，鼓励创业带动就业。加大政策扶持和财政投入力度，认真落实税费减免、社保补贴、就业服务补贴、国有大中型企业主辅分离辅业改制安置富余人员等优惠政策，完善再就业援助制度，着力帮助就业困难群体特别是“零就业”家庭、“４０５０”人员和残疾人就业。改善进城务工人员的就业环境，建立健全劳动关系调整机制，促进劳动关系和谐。

加快完善社会保障体系，保障人民群众的基本生活。进一步完善城镇职工养老保险制度，扩大养老保险覆盖面。按照国家、集体、个人三方负担的原则，逐步建立被征地农民的养老保险制度。逐步建立与现行养老保险制度相衔接的农民工养老保险办法。按照国家和省的统一部署，进一步扩大失业、医疗保险覆盖面。加强公共卫生和基本医疗体系建设，努力解决群众看病难、看病贵的问题。规范和加强经济适用房建设，加快廉租房建设，逐步解决城镇中低收入家庭住房困难。进一步完善城乡低保制度，继续加强城镇困难居民和农村特困群众大病救助，适度扩大救助范围，提高救助标准。加大政府投入，切实加强农村基层文化教育卫生工作，加强农村学校危房改造和教师队伍建设。提高乡镇卫生院建设水平，加强行政村卫生所建设，基本建立新型农村合作医疗。加强血吸虫疫区的水环境治理，有效预防和控制血吸虫病。坚持做好扶贫、救灾等工作，继续做好深山库区移民搬迁扶贫工作，大力发展慈善事业、残疾人事业和老年人事业。

加强社会管理，维护社会安全稳定。进一步强化政府的社会管理和公共服务职能，创新公共服务体制，改革公共服务方式，加强公共设施建设。全面加强城市社区建设和管理，大力发展社区服务，积极推进农村村落社区建设。进一步加强和改进政府信访工作，健全信访工作责任制。健全社会舆情汇集和分析机制，完善矛盾排查调处工作制度。提高危机管理和抗风险能力，建立健全应急管理机制，形成统一指挥、反应灵敏、协调有序、运转高效的政府应急管理机制，建立完善赣北地震监测中心，有效应对自然灾害、事故灾难、公共卫生事件和社会安全事件。高度重视安全生产，以道路交通、易燃易爆、煤矿和非煤矿山、食品药品安全为重点，坚决遏制重特大安全事故发生。依法严厉打击各种严重犯罪活动，综合治理社会治安突出问题，扎实做好“扫黄打非”工作，保持社会安定、环境安全、百姓安宁。

三、二００七年工作安排

２００７年是新一届政府工作的开局之年，开好头、起好步，对于实现今后五年的奋斗目标和工作任务至关重要。明年经济和社会发展的主要预期目标是：全市生产总值５７６．８亿元，增长１４％，财政总收入５２．９亿元，增长１５％，地方一般预算收入２８．２亿元，增长１６％，规模以上工业增加值增长２０％，全社会固定资产投资增长３０％，社会消费品零售总额增长１４％，城镇居民人均可支配收入增长１０％，农民人均纯收入增长８％，城镇登记失业率控制在４％以内，人口自然增长率控制在７．６９‰以内，单位ＧＤＰ能耗下降４％。为实现上述目标，重点要抓好六个方面的工作：

（一）加大对外开放力度，提高开放型经济发展水平

全力推进招商引资。有针对性开展招商活动，继续承办赣台经贸合作研讨会，举办新加坡、香港经贸洽谈会，组织欧洲、香港、上海、福建、宁波、东莞等地的招商活动。充分发挥新加坡、台湾、福建、浙江、广东五大商会的作用，以商招商。完善政策措施，提高引资质量，推行项目链、产业链、客商链招商，力争在工业、农业、城建、旅游、商贸等领域引进一批带动能力强、科技含量高的大项目，提高利用外资的质量和水平。建立项目推进督查机制，实行大项目领导挂钩、专人负责制，限定时间、跟踪落实，推进武藏野聚合乳酸、波兰直升机、思麦博运动器材、鹿角尖国际会议中心、挪宝电器、赛得利二期、亚泥三期、培罗成服装、赣北钨业ＡＰＴ二期建设，促成星火有机硅氟化工再上３０亿以上投资项目，凤竹纺织二季度竣工投产。努力降低招商成本，规范用地，节约用地，有效破解土地、资源等要素约束难题，提高履约率和投资强度。力争实际利用外资３．８亿美元，引进市外资金增长１５％。

努力扩大外经外贸。坚持以质取胜、科技兴贸，推进外贸增长方式转变，扩大外贸出口规模，推动特色工业品和优质农副产品采用国际标准，参与国际市场竞争。设立外经外贸发展促进专项基金，组建外贸资产管理公司，增强外贸企业出口创汇能力。大力扶持财兴五金等出口骨干企业做大做强。帮助更多私营企业获得出口经营权，鼓励造船、机电产品出口和加工贸易。力争全年外贸出口增长１５％。进一步做好外事、侨务和对台工作。

改善开放服务功能。加强口岸建设，完善出口通关的各项设施，为外贸出口企业提供高效便捷服务。大力发展涉外中介机构，进一步加强涉外人才队伍建设，大力发展面向境内外的会展经济。打造对接大通道，为交通重点工程建设提供有力的保障服务。

（二）狠抓工业园区建设，增强产业集聚和配套能力

大力发展园区经济。精心谋划推进沿江和昌九沿线产业布局，加大政策扶持、用地优先、资金倾斜、人才支撑的力度，加快路网、电网、水网等各项基础设施建设，扶持九江经济技术开发区、共青开发区，做大做强出口加工区、综合工业园、码头工业城、金砂湾工业城，培育有机硅、电子电器、汽车配件、船舶制造、重化工等特色工业园，形成工业迅速扩张的核心增长极。突出项目建设，加强市县联动，鼓励多元开发，确保每县都有１∽２个亿元以上的项目落户工业园，促进产业集聚和产业配套，形成项目集中、产业集聚的经济板块。

培植壮大支柱产业。围绕支柱产业的发展，着力抓好年销售收入过亿、利税过亿、投资过亿的项目，抓好博升钢铁（萍钢）技改扩能，扶持昌铃汽车、卡博特、九江巨石、华孚纺织等企业提升产能，促进东海造船、之江化工、新康达离子膜烧碱、回园服装等项目竣工投产。力争规模以上企业达５５０户，销售收入过亿元企业达７５户。同时，尽快出台支持民营经济发展的实施意见，加大政策扶持力度，建立民营经济发展专项资金，规范担保办法，促进中小企业发展，推动产业协作配套，提升工业竞争力。

大力支持国家和省属企业的发展。推行高位引进、高位重组，抓好与江铜的全面合作，促使中国五矿新上深加工项目，跟踪落实与中国化建集团合作项目，促进中石化增加投资扩大产能，主动对接清华同方在我市兴建新的生产基地。深入推进企业改制，完善配套政策，支持配合九化、化纤、中船等驻市企业改制，确保完成江氧等１０户企业改制任务。充分发挥各种所有制的骨干企业对地方经济的带动和支撑作用。全市规模以上工业增加值、销售收入、利税总额分别增长２０％以上。

（三）提高农村经济发展和农民致富的水平，切实改善农民生产生活条件

加强农村基础设施建设。加大政府公共财政投向农村基础设施建设的力度，整合农业开发、水利、移民、扶贫、交通、文化、卫生等专项资金，引导工商资本、民间资本投入新农村建设，加快基础设施向农村延伸、公共服务向农村覆盖、现代文明向农村辐射。全市争取省级试点村１０００个，并按每个行政村抓好一个自然村建设点的要求，以搞好村庄规划和改水、改厕、改路为着力点，加强村容村貌整治。因地制宜搞好小城镇规划。全年完成农村油（水泥）路１５００公里、砂石路８５０公里，启动３１６国道改造项目，实现３００个行政村通油（水泥）路、１７０个行政村通公路。实施农村饮水安全工程，改水改厕６万户，新建农村沼气池２．５万个，争取实现３００个自然村广播电视“村村通”。

加快现代农业建设。加快农村经济结构调整，加强优质农产品基地建设，继续抓好粮食、棉花、油菜等大宗农产品生产，大力发展早熟梨、蚕桑、蔬菜、药材、油茶、花卉苗木和规模经济林，推动生猪、水产等特色养殖，培育“一乡一业”的产业优势。扶持鄱湖水产、汇源果汁、仙客来食品、绿冬丝科、博莱兽药等产业关联度高、市场竞争力强的龙头企业发展，提高农产品精深加工水平。全市重点扶持４０个经济关联度高的农民专业经济合作组织，培养农民经纪人队伍，探索提高农民组织化程度新路径。加强科技兴农，加快农业技术推广和农产品标准化步伐，提高农民的整体素质。加快实施“阳光工程”和“农民知识化”工程，全年培训转移农村劳动力２万人以上，组织２０万人参加农业实用技术培训。

提高农业农村持续发展能力。巩固农村税费改革成果，推进农村综合改革。认真抓好农村水利建设，实施“百库除险，千渠疏浚，万塘整治”，启动１０座中型水库除险加固建设，完成４０座小Ⅰ型水库除险加固，抓好９个灌区水利血防工程建设，完成水土流失综合治理面积３０万亩。加强气象、水文及预警预报系统建设，建立完善防汛抗旱减灾体系。认真抓好２０９个扶贫开发重点村规划项目的实施。

（四）壮大旅游物流，提升现代服务业水平

提升重点旅游景区的品质和效益。组织实施市、县两级旅游发展总体规划，编制重点旅游景区的控制性详规，搞好历史文化街区、风味饮食一条街、旅游购物步行街和旅游商品交易市场等专项规划。更好地发挥庐山旅游龙头作用，力争庐山成功申报国家５Ａ级旅游景区、云居山－柘林湖和星子温泉成功申报国家４Ａ级旅游景区，继续做好庐山山门下迁工作，推进庐山居民山下住宅区建设，完善共青高尔夫球场建设。加强与国际国内旅游运营商的战略合作，重点引进香港中旅、中国国旅、首都旅游等知名集团加盟九江旅游。把文化底蕴与饮食、歌舞、民俗、传奇等结合起来，着力打造南线宗教文化山水游、东线名湖名洞游、西线红色游、农家休闲游等精品线路。加大入境旅游和国内大城市及远端客源市场的营销力度，办好九江（庐山）国际旅游节和中国（九江）国际龙狮精英赛。做优砚台、蚕丝、珍珠、剪纸等特色产品，打响“浔阳八珍”、“吉祥三宝”品牌。

大力改善商贸物流发展的基础条件。抓紧制订物流发展规划，完善物流发展政策，促进行业、部门、区域合作。加快湖口港口物流园区、九江综合物流园区建设，搞好昌铃汽车、石化产品和邮政等１０大物流中心建设，扶持京九农副产品批发、粮食交易等１０大中心市场做大做强。力促沃尔玛早日建成。坚持新建和扩张并举，科学规划、合理开发，大力发展综合性码头，加快２１４码头改造，扶持民航机场正常运行。港口集装箱吞吐量力争突破１０万标箱，确保全社会货运总量增长２０％。

加快发展现代服务业。出台支持服务业发展的实施意见，整治社会信用环境，培育信用中介机构，构筑相依、互信、双赢的新型银政、银企关系，加大对民营企业、农村经济和服务业的信贷支持，优化消费信贷，增强防范金融风险能力。加快发展律师、会计、审计等中介机构。建立以电子政务、电子商务、电子社区为基础的综合信息服务体系，办好“中国九江网”，启动“政务直通车”信息系统建设。全面整顿和规范房地产交易秩序，培育公开透明、健康发展的房地产交易市场。积极发展文化创意产业、城市宾馆休闲餐饮娱乐业。

（五）完善城市功能，努力构建最适宜的人居环境

加强城镇规划编制。更新城镇发展理念，继续完善现有规划成果。全面完成中心城区控制性详规，县城控规覆盖率达８５％，村庄规划编制率达６０％以上。

完善城市承载功能。加快城区道路交通、市政管网、园林绿化、环境保护、公共服务、旅游休闲等设施建设，提高城市综合承载力。实施并完成鹤问湖环湖大道，启动该区域的开发建设和十里濂溪芳兰三个组团、综合工业园、汽车工业园主路网建设，大力实施经济适用房、廉租房建设。完成滨江路截污、体育中心、疾控中心等续建项目。启动十里河整治、博物馆、鹤问湖污水处理厂、第二垃圾处理场、长虹东路、濂溪大道等项目。进一步加强对县城建设的指导和支持。

提高城市管理水平。健全城市管理的长效机制，完善统一领导、属地管理、部门协调、权责分明的城市管理机制。继续开展市容环境综合整治，切实提高执法队伍素质，做到公正、文明执法。对市政公共事业逐步实行市场化运作、企业化经营、社会化服务。创建全国文明卫生城市和园林城市。

（六）完善公共服务体系，统筹发展社会各项事业

突出发展教育和科技事业。强力推进我市“十一五”科技发展规划的实施。深化科研机构和科技评价、奖励制度改革，建立高校与地方互动机制，发挥高校人才优势，加强生物医药、微电子、工程机械等重点领域的关键技术攻关，集中力量在应用领域实现新的突破。实施农村中小学设施改造工程。做强做大九江学院，推进九江工程学院、江西财经职业技术学院、九江职业大学、旅游中等专科学校建设，在校大学生达到１０万人。发展出版发行、广播影视等主导文化产业和会展、广告、演艺等社会文化产业，普及乡镇文化站，电视人口覆盖率达９９％。做好农业普查工作，启动第二轮市志修编。积极发展体育事业，实现群众体育与竞技体育共同提高。

切实加强人口、就业和社会保障工作。坚决执行生育政策，稳定低生育水平。加强基层计生服务网络建设，努力解决出生人口性别比失衡问题。坚决兑现各类扶持就业和再就业政策，在资金、技术、场地等方面给予最大方便和优惠，继续搞好“零就业”家庭的帮扶，对有就业愿望的城乡劳动力免费提供就业培训。全年新增城镇就业５万人，新增转移农村劳动力５万人。进一步扩大社会保险覆盖面，加强基金管理，提高备付能力。进一步完善城乡最低生活保障制度、城乡医疗救助制度和疾病控制体系。新型农村合作医疗试点县达７０％。构建公共财政体系，加大对弱势群体和社会保障的支出，让群众共享改革发展的成果。

扎实做好和谐稳定的基础工作。建立政府问责制，完善安全生产和稳定目标管理与考核机制，加强安全执法和责任追究。实施食品、药品质量安全市场准入制度，强化食品、药品安全体系建设。完善政府领导干部接待群众制度，在乡镇、街道建立矛盾排查调处中心，切实把矛盾化解在基层、解决在萌芽状态，有效预防和妥善处置群体性事件。坚持“打防结合、预防为主、专群结合、依靠群众”的方针，深化平安县（市）、平安乡村、平安社区、平安校园等基层平安创建活动，切实把社会治安综合治理的各项措施落实到基层。增强国防意识，加强国防后备力量建设。

在完成上述工作的同时，重点为全市人民办好十件实事：

１、实现城乡困难群众低保和大病医疗救助全覆盖，帮助２０００户“零就业”家庭就业，扶持１０００名残疾人就业；

２、免除全市城乡义务教育学杂费，公开招聘２０００名以上乡村教师，着力解决农村学校师生比失衡问题；

３、完成廉租房二期工程，解决４００户特困家庭住房困难，建成２０万平方米的经济适用住房，改善中低收入家庭住房条件；

４、完善市政公共生活设施配套，方便群众生活，在市区重要场所新建２０个水冲式公厕，６个垃圾中转站，每一个街办辖区新建一个农贸市场；

５、新增农村安全饮水人口５万人，解决５万农村人口饮水困难问题；

６、搬迁深山移民９０００人，改善边穷地区群众生产生活条件；

７、启动九江化工厂的搬迁和鹤问湖污水处理工程，逐步改善中心城区环境质量；

８、建成公路客运中心和公交总站，解决城乡出行畅通问题；

９、启动建设公共服务中心，整合供水、供电、救灾、救护等部门资源，为市民提供集约型、综合性的优质服务；

１０、扶持扩大蔬菜基地建设，管理搞活流通市场，认真解决城区居民买菜难、吃菜贵的问题。

四、加强政府自身建设

新起点、新形势、新任务，对政府工作提出了更高的要求。要将今后五年的宏伟蓝图变为美好现实，实现九江经济社会又好又快发展，迫切需要各级政府强化自身的改革和建设，努力打造“亲民为民、创新求进、诚信务实、高效廉洁”的政府，不断提高执政能力和水平。

坚持开拓创新，不断增强科学发展能力。各级政府和全体政府工作人员要贯彻落实科学发展观，始终抓住发展这个第一要务，增强加快发展的责任意识、忧患意识。努力提高领导发展、落实发展、服务发展的能力。通过加强学习，掌握市场经济知识和社会管理知识，增强贯彻落实中央、省委、省政府和市委方针、政策、决策的坚定性和领悟力。把握国际国内发展趋势，善于科学分析和判断经济社会发展形势，以现代开阔的工作视野，把握机遇，应对挑战，加快发展。大力提高开放搞活的创新能力。保持奋发有为的精神状态，进一步解放思想，摒弃观念桎梏，突破体制障碍，以政策的开明带动社会各方面的开放，以机制创新促进各行各业的能量释放。切实提高落实决策的执行能力。市委提出的正确思路能否贯彻，美好蓝图与目标能否实现，关键在于政府的执行力。各级政府和各级干部要树立强烈的大局意识，坚决反对“政府权力部门化，部门权力利益化”，坚决防止 “上有政策，下有对策”，坚决防止政令不畅，坚决防止落实工作只抓其表、不抓其实，切实提高政府抓落实的水平。不断提高破解难题的操作能力。以止于至善的工作要求，切实提高工作的开拓性和坚韧性，对看准了的事情要不变频道，不换镜头，真抓实干，迎难而上，一以贯之，在处理复杂矛盾中不断提高解决实际问题的能力和水平。

切实履行责任，倾注心力为民服务。各级政府和全体政府工作人员要牢固树立执政为民的理念，切实履行工作职责。必须时刻牢记人民政府为人民，心里没有群众就是忘本。把实现维护发展群众利益当作根本，认认真真访民情，诚诚恳恳听民意，真真切切解民忧，实实在在帮民富，兢兢业业保民安，努力提高为人民服务的水平。为群众服务贵在求实，坚决防止和克服形式主义和官僚主义，大兴求真务实之风，从群众反映最强烈的问题抓起，从群众最不满意的地方改起，从群众最企盼的事情做起，从柴米油盐、衣食住行、入学就业、求医问药、助老托幼等方面入手，尽心竭力办好事、办实事，为百姓遮风挡雨，替群众排难解忧。在高速发展同时，加大对关系民生的设施投入，真正做到向困难群体倾斜、向基层倾斜、向社会事业倾斜；在城市建设中，更多地考虑方便市民的工作生活；在社会管理时，更多地体现人文关怀，既要坚持依法行政，又要寓管理于服务之中，在服务中体现管理。

完善决策机制，提高科学民主决策水平。把科学民主决策作为政府自觉追求和基本制度，合理界定政府的决策权限，完善科学民主决策机制，进一步健全政府重大决策的调查研究、公众参与、专家论证、社会公示、听证旁听、决策评估等制度，重大问题注重听取市人大、市政协意见，征求党委意见并报备。提高决策的民主化、科学化、法制化水平，使决策更具实际效果和可操作性。各级政府要自觉接受人大及其常委会的依法监督，把依法行政作为政府工作的基本准则。接受人民政协的民主监督，加强与人民政协的联系和协商。

创新完善运行机制，提高政府工作效能。按照提质、提速、提效的要求，加快建立办事高效、运转协调、行为规范的行政管理运行机制，努力建设效能政府。继续推进政企分开、政事分开和政社分开，把政府职能转移到经济调节、市场监督、社会管理和公共服务上来，运用经济手段和法律手段管好市场秩序、法制环境，防止政府行为缺位。继续深化行政审批制度改革，加快对行政服务中心的办批授权，规范政府工作流程，简化办事程序，提高行政效率，推动工作全面提速。发扬立说立干、雷厉风行的工作作风，对市委作出的重大决策，做到反应迅速、推进有力、落实到位。深入开展优化发展环境机关效能监察，采取有效措施，坚决纠正行政不作为、行政乱作为、损害群众利益、破坏发展环境等行为，推进依法行政，科学施政，廉洁从政。认真贯彻实施《公务员法》，强化教育，严格管理，切实提高公务员队伍素质。

加强廉政建设，严格规范从政行为。坚持从严治政，切实贯彻执行党风廉政建设责任制。组织专门力量研究建立健全结构合理、配置科学、程序严密、制约有效的权力运行机制。加快市级统一的公共资源交易中心建设，规范建设工程招投标、经营性土地使用权出让、政府采购和产权交易行为，加强权力制约，规范事权财权，加强审计监督。规范各级政府和公务员从政行为，政府领导成员要带头廉洁自律，严格遵守各项规章制度，真正做到能干事、干实事、干成事、不出事。强化社会监督，切实纠正部门和行业不正之风。各级政府要牢固树立艰苦奋斗、勤俭节约的思想，坚持珍惜民资民力，坚决反对挥霍奢侈、铺张浪费，以廉洁的政府形象取信于民。

各位代表，在新起点上实现九江经济社会又好又快的发展，是时代赋予我们的神圣使命，是我们共同的奋斗目标。让我们紧密团结在以胡锦涛同志为总书记的党中央周围，高举邓小平理论和“三个代表”重要思想伟大旗帜，全面贯彻落实科学发展观，在省委、省政府和市委的领导下，一届接着一届干，紧紧团结和依靠全市人民，齐心协力，奋发有为，为了九江人民的福祉，为了九江光辉灿烂的明天而努力奋斗！

关联导读：

附件：
